# Gut Microbiota and Environment in Coronary Artery Disease

**DOI:** 10.3390/ijerph18084242

**Published:** 2021-04-16

**Authors:** Andrea Piccioni, Tommaso de Cunzo, Federico Valletta, Marcello Covino, Emanuele Rinninella, Pauline Raoul, Christian Zanza, Maria Cristina Mele, Francesco Franceschi

**Affiliations:** 1 Department of Emergency Medicine, Fondazione Policlinico Universitario A. Gemelli IRCCS, Università Cattolica del Sacro Cuore, Largo A. Gemelli, 8, 00168 Rome, Italy; tomdecunzo@gmail.com (T.d.C.); fede.valletta@gmail.com (F.V.); marcello.covino@policlinicogemelli.it (M.C.); christian.zanza@live.it (C.Z.); francesco.franceschi@unicatt.it (F.F.); 2 UOC di Nutrizione Clinica, Dipartimento di Scienze Mediche e Chirurgiche, Fondazione Policlinico Universitario A. Gemelli IRCSS, 00168 Rome, Italy; emanuele.rinninella@unicatt.it; 3 Dipartimento di Medicina e Chirurgia Traslazionale, Università Cattolica del Sacro Cuore, 00168 Rome, Italy; pauline.raoul1@gmail.com (P.R.); mariacristina.mele@unicatt.it (M.C.M.); 4 UOSD di Nutrizione Avanzata in Oncologia, Dipartimento di Scienze Mediche e Chirurgiche, Fondazione Policlinico Universitario A. Gemelli IRCSS, 00168 Rome, Italy

**Keywords:** gut microbiota, coronary artery disease, trimethylamine-N-oxide, short-chain fatty acids, lipopolysaccharides, pollution, Western diet

## Abstract

In recent years, studies evaluated the associations between coronary artery disease (CAD) and fecal gut microbiota composition. This opens new perspectives on therapeutic strategies to prevent CAD representing the leading cause of mortality in Western societies. We have conducted a review of the literature regarding the characteristics of the gut microbiota of CAD patients, its underlying mechanisms and their associations with pollution and the Western diet. The latest evidence confirms that an abnormal microbiota predisposes to the development of CAD and differs in composition compared to the microbiota of healthy patients; the results are, however, heterogeneous. The most studied underlying mechanisms involve the production of trimethylamine-N-oxide (TMAO), the synthesis of short-chain fatty acids (SCFAs) and the immune system activation mediated by lipopolysaccharides (LPS). Despite a large amount of available data, there is no evidence about the role of a specific type of gut microbiota in the risk of developing acute coronary syndrome (ACS). Moreover, no relationship has been assessed between the gut microbiota and the characteristics of coronary plaques in humans. However, a close association has been found between both pollution and the Western diet and gut microbiota and CAD. Further studies are needed to clarify the associations between gut microbiota, CAD, and ACS to find efficient therapeutic strategies.

## 1. Introduction

Coronary artery disease (CAD) is the leading cause of mortality in Western societies, affecting about one-third of the population before the age of seventy [1]. Over the past decades, several risk factors for CAD have been identified, including smoking, hypertension, hypercholesterolemia, diabetes, and obesity [2]. Despite the increasing ability to correct such factors, the incidence of heart diseases remains high and they still represent the major cause of mortality. Therefore, research has recently focused on finding new risk factors so as to identify alternative therapeutic strategies and prognostic indexes. Among these, the gut microbiota.

With a total of more than 10^13^ microorganisms, gut microbiota refers to the entire population of bacteria, archaea, viruses, and protozoans that colonize the intestinal tract, most of them within the large intestine, where there is over than 70% of all microbes found in the body. The predominant phyla are Firmicutes and Bacteroidetes followed by Proteobacteria and Actinobacteria. There are also pathogenic species such as *Campylobacter jejuni, Salmonella enterica, Bacteroides fragilis, Vibrio cholera,* and *Escherichia coli*, but in a low abundance (0.1% or less) [3]. *Bacteroides, Bifidobacterium, Streptococcus, Enterobacteriaceae, Enterococcus, Clostridium, Lactobacillus,* and *Ruminococcus* are the predominant luminal microbial genera and they can be identified in stool [4].

Gut microbiota strongly varies depending on several environmental and lifestyle factors, such as pollution and diet [5,6,7], and maintains a symbiotic relationship with the gut mucosa, with substantial metabolic, immunological, and gut protective functions in the healthy individual [8,9,10,11].

In recent years, diseases such as inflammatory bowel disease [12], asthma [13], depression [14], and Hashimoto’s thyroiditis [15] have been associated with the composition of the gut microbiota. However, research has also focused on heart diseases, and gut microbiota has been shown to affect the cardiovascular system through different mechanisms, representing a potentially modifiable risk factor for atherosclerosis. This opens new perspectives on therapeutic and preventive strategies for CAD.

The hypothesis that microorganisms can influence coronary atherosclerosis was first proposed a long time ago, when, in 1978, Fabricant et al. demonstrated that Marek’s herpes virus could cause atherosclerosis in chickens [16]. They subsequently observed that such an atherosclerotic effect could be prevented by vaccination, thus revealing the therapeutic potential of these experiments [17]. Since then, many microorganisms have been associated with coronary atherosclerosis, and some of them are also thought to act with a direct mechanism: the presence of bacterial deoxyribonucleic acid (DNA) in human atherosclerotic plaques has been demonstrated, especially of microorganisms from the oral cavity [18,19] or the respiratory tract [20]. In other cases, microorganisms such as HIV or Helicobacter Pylori can cause chronic inflammation that predisposes to the development of atherosclerosis [21,22].

In this review, we provide an update of the literature about the associations between gut microbiota and cardiovascular disease, focusing on CAD and highlighting possible associations of pollution and diet, microbiota and CAD.

## 2. Methods

A literature research was performed in PubMed and Google Scholar electronic databases, using the following keywords: “gut microbiota”, “atherosclerosis”, “coronary artery disease”, ST elevation myocardial infarction (“STEMI”), “metabolism”, “inflammation”, “trimethylamine N-oxide”, “TMAO”, “SCFA”, “LPS”, “pollution”, “diet”, “carnitine”, “meat”, “choline”. Only English-language articles were included, and preference was given to papers published within the last 10 years. We have searched the bibliographies of the selected articles to identify other relevant articles. We have excluded studies over 10 years old, inappropriate or not relevant topics to the specific focus of this review.

## 3. Results

Forty-five studies assessing the associations between gut microbiota and cardiovascular disease were included. Table 1, Table 2, Table 3 and Table 4 describe the study design, year of publication, subjects evaluated and the main findings.

Table 1 summarizes nine studies [23,24,25,26,27,28,29,30,31,63] evaluating the gut microbiota composition in CAD patients. Most of them are comparative studies [24,25,26,28,29,30,31,63]. All studies found that the microbiota composition of CAD patients differed from that of healthy controls, although the results are not always overlapping and potential causal relationships are challenging [64].

Table 2 details twenty-one studies [32,33,34,35,36,37,38,39,40,41,42,43,44,45,46,47,48,49,50,51,52] assessing the association between atherosclerosis and TMAO synthesis. A TMAO increase was found in patients with cardiovascular disease. Prospective studies in mice [41,42,43,44,45,46,48,49,50,51,52] have shown that TMAO has proinflammatory, prothrombotic and dyslipidemic effects.

Table 3 and Table 4 summarize, respectively, four and six studies reporting the association of SCFAs and LPS with gut microbiota. SCFAs play a protective role against atherosclerosis while LPS activates systemic inflammation and accelerates the formation of the atherosclerotic plaques.

Table 5 details five studies about the connection between pollution and gut microbiota. Those point out that pollution can influence cardiovascular disease despite changes in the microbiota, opening new perspectives on prognostic and therapeutic strategies for these diseases.

Finally, Table 6 details the studies assessing the relationship between microbiota, diet and CAD.

## 4. Gut Microbiota Composition in CAD Patients

Considering these observations, in recent years, research has been focusing on the association between coronary atherosclerosis and gut microbiota composition. Although a close relationship between gut microbiota and atherosclerotic plaque formation has not yet been demonstrated, the latest evidence confirms that an abnormal microbiota predisposes to the development of CAD [23] and that gut bacterial composition differs in CAD patients compared with healthy patients; the results are, however, not homogeneous in terms of specific differences. A study analyzed fecal metagenomes of 12 patients with symptomatic atherosclerotic plaques showing an increase in the abundance of *Collinsella* and a reduction in the abundance of *Eubacteria*, *Roseburia* and Bacteroides species compared with the control group [24]. Emoto et al. reported an increase in *Lactobacillales* and a reduction in the abundance of Bacteroidetes phylum (*Bacteroides* + *Prevotella*) in CAD patients [25]. Another comparative study conducted by Zhu et al. reported a significant enrichment of *Faecalibacterium*, *Subdoligranum*, *Roseburia* and *Eubacterium*, while *Escherichia*, *Shigella* and *Enterococcus* abundances were reduced in CAD patients, hypothesizing that this bacterium plays an anti-inflammatory role [26]. A long-term epidemiologic study, using collected measures of systolic blood pressure, LDL cholesterol, and glucose to establish the cardiovascular risk, revealed that the growth of *Prevotella 2, Prevotella 7, Tyzzerella* and *Tyzzerella 4* were associated with high cardiovascular disease risk while *Alloprevotella* and *Catenibacterium* were associated with low risk [27]. Li et al. found an overall reduction of microbial richness and diversity, a depletion in abundances of *Faecalibacterium*, *Oscillibacter*, *Roseburia*, *Bifidobacterium*, *Coprococcus*, and *Butyrivibrio* and an overgrowth of bacteria such as *Prevotella* and *Klebsiella* in patients with primary hypertension, one of the most important risk factors for CAD [28]. More recent studies used the 16S ribosomal RNA sequencing for the identification of operational taxonomic units (OTUs) through which microorganisms are classified and then identified [63]. Using this technique, Cui et al. observed a decrease in Bacteroidetes and Proteobacteria phyla in CAD patients, whereas the phyla Firmicutes and Fusobacteria were increased [29]. Yoshida et al. reported the reduction of *Bacteroides vulgatus* and *Bacteroides dorei* in CAD, assuming that this is due to a higher systemic inflammatory effect that in healthy patients is inhibited by these two species [30]. Toya et al. showed a reduction of overall bacterial richness and evenness, an increase in *Ruminococcus gnavus* abundance, and a depletion in *Lachnospiraceae NK4B4* and *Ruminococcus gauvreauii* levels in the gut microbiota of CAD patients [31].

## 5. TMAO, Microbiota, and CAD

One of the most studied mechanisms behind the association between gut microbiota and cardiovascular diseases involves TMAO. When large quantities of choline, carnitine, betaine, and other choline-containing compounds are ingested, intestinal bacteria degrade them into trimethylamine (TMA) which passes into the portal circulation and is then metabolized in TMAO by flavin-containing monooxygenase (FMO) enzyme in the liver [64]. Several studies have shown a direct correlation between TMAO levels and coronary atherosclerosis [32,33]. In fact, TMAO has a positive correlation with metabolic syndrome [34] and with age, BMI, total and LDL cholesterol, apolipoprotein B levels and tumor necrosis factor (TNF) alpha levels [35]. TMAO increases the risk of ventricular arrhythmia in stroke patients [36]. Even the most recent studies have confirmed the use of TMAO levels as a prognostic marker for cardiovascular disease [37,38]. In a prospective study conducted on 60 patients with STEMI or unstable angina, Gan et al. showed a direct correlation between TMAO levels and acute coronary syndrome (ACS) [39]. Tascanov et al. confirmed this association in a cohort of young ACS patients (age < 50 years) [40].

The mechanism through which TMAO promotes atherosclerosis is not yet known, but studies suggest that inflammation plays a central role. Seldin et al. found an activation of nuclear factor-κB (NF-κB) signaling and an enhancement of endothelial recruitment of leukocytes [41]. Ma et al. confirm these results and suggested that TMAO up-regulates vascular cell adhesion molecule-1 (VCAM-1) expression and promotes monocyte adherence [42]. Chen et al. demonstrated that TMAO promotes vascular inflammation in mice by activating nucleotide-binding oligomerization domain–like receptor family pyrin domain containing 3 (NLRP3) inflammasome [43]. TMAO also increases the expression of CD36 via the MAPK/JNK pathway, thus promoting atherosclerosis [44].

In addition to the inflammatory mechanism, in recent years, a metabolic role of TMAO has also been investigated. TMAO reduces the reverse cholesterol transport, through which cholesterol is transported from peripheral tissues to the liver and small intestine, and increases macrophage scavenger receptor A and CD36 surface expression and foam cell formation [45,46]. In a trial conducted by Chen et al., the administration of resveratrol to mice inhibited TMAO production, enhancing bile acid neosynthesis in the liver [47]. In a more recent study, Ding et al. found an activation of nuclear receptor farnesoid X receptor (FXR) and small heterodimer partner (SHP) mediated by TMAO that suppresses CYP7A1 expression and ultimately inhibits bile acid synthesis, increasing fat mass, serum lipid concentrations and atherosclerosis [48].

TMAO may also have a prothrombotic effect. In a murine model proposed by Zhou et al., it promotes platelet responsiveness by enhancing the stimulus-dependent release of Ca2+ from intracellular Ca2+ stores [49]. These observations have been confirmed by a trial conducted by Skye et al. in which a transplant of human feces with a prothrombotic phenotype to a germ-free mouse was performed [50]. Shih et al. has recently proved that lower TMAO levels observed in FMO3 knockout mice were accompanied by decreased platelet responsiveness [51]. Roberts et al. suggested the use of drugs that inhibit TMAO to reduce thrombotic potential [52].

## 6. SCFAs, Microbiota, and CAD

Gut microbiota can also play a protective role against coronary atherosclerosis through the synthesis of SCFAs. SCFAs are the main products of intestinal fermentation of dietary fibers, and acetate, propionate, and butyrate are the most abundant [77]. These SCFAs can bind to a lot of G-protein coupled receptors (GPR) and may have an anti-inflammatory and immunomodulatory effect depending on this bond. Acetate is produced by many intestinal bacteria while only a few members of the families *Veillonellaceae* and *Lachnospiraceae* are propionic acid-producing bacteria, and butyrate is synthesized by *Coprococcus*, *Faecalibacterium*, *Eubacterium*, and *Roseburia* [78]. McNelis et al. demonstrated that SCFAs in mice protect against diet-induced-obesity and improve glucose and lipid metabolism through the bond with GPR43/FFAR2 and GPR41/FFAR3 [53]. Another experiment conducted on mice by den Besten et al. showed that butyrate and propionate reduce body weight and liver triglyceride accumulation through activation of peroxisome proliferator-activated receptor-γ (PPARγ) [54]. Vinolo et al. showed a general reduction of cytokine release mediated by SCFA [55], and Aguilar et al. found a reduction of atherosclerotic plaque in mice after oral butyrate administration [56]. Although all these studies highlight the protective role of SCFA on atherosclerosis and inflammation, human studies are still lacking.

## 7. LPS, Microbiota, and CAD

Gut microbiota can trigger the immune system and atherosclerosis through Toll-like receptor (TLR) activation by LPS. LPS, also called endotoxin, is a Gram-negative bacteria membrane component and its correlation with the atherosclerotic process has been demonstrated long ago in experimental mice models [57], through induction of a low-grade inflammation [58]. A recent metagenomic analysis conducted by Zhou et al. has compared 100 STEMI patients with 49 healthy control and 50 stable CAD subjects, proving that the production of LPS increases in the first group and assuming that it may be due to a raise in intestinal permeability [59]. Kasahara et al. hypothesized that the reduction of atherosclerosis in germ-free mice, despite a significant increase in the plasma and hepatic cholesterol levels, is related to the absence of LPS [60]. Even in this case, however, the data are contradictory and many studies, despite suggesting an increase in inflammation, have failed to demonstrate a statistically significant correlation between LPS and atherosclerosis [61,62].

## 8. Pollution, Microbiota and, CAD

It is well known that one of the most important risk factors for cardiovascular disease is environmental pollution, meaning gas emission of chemical contaminants [79]. This is mainly because exposure to environmental pollutants promotes a systemic vascular oxidative stress reaction and a rise of radical oxygen species which induce endothelial dysfunction, monocyte activation and changes in lipoproteins [80]. Recent studies are revealing how pollution not only directly affects the composition of gut microbiota but also regulates its interaction with the immunity system and production of metabolites involved in atherosclerosis [81].

Exposure to heavy metals is associated with a large number of toxic effects, even on the cardiovascular system [82]. Different studies observed that mice exposed to cadmium have significant changes in gut microbiota, an increase in LPS production and an alteration in bile acid formation [65,66]. Some pesticides that contaminate food, water and soil can induce a gut dysbiosis that promotes a pro-inflammatory state and metabolic disorders: exposure to carbendazim, a broad-spectrum benzimidazole fungicide, changes gut microbiota composition and reduces the level of serum lipoprotein lipase, altering lipid metabolism [67]. Particulate matter air pollution, a combination of elements, heavy metals, polycyclic aromatic hydrocarbons and inorganic ions [83], has been associated with a spectrum of disease which goes from lung cancer to hypertension [84,85] and can also affect the gut microbiota, leading, again, to an increase in inflammatory response as revealed in studies conducted on murine models [68,69].

## 9. Diet, Microbiota, and CAD

Gut microbiota is involved in the metabolism of dietary sources and rapidly varies according to dietary habits [86]. A growing number of animal and human studies [45,70,72,73,74,75,87,88] have reported a close relationship between dietary patterns, gut microbiota, the formation of microbiota-derived metabolites such as TMAO and the development of cardiovascular diseases (Table 6).

In more than 4000 patients undergoing coronary angiography, a strong association was found between fasting plasma levels of TMA and the incidence of adverse cardiovascular events [73]. As previously described, TMA, rapidly oxidized into TMAO, is a gut microbe-dependent metabolite mainly generated from dietary choline and L-carnitine. These dietary components are found in a high variety of foods. The richest sources are meat, fish, poultry, dairy, and eggs, representing predominant food groups of a Western diet. Moreover, elevated TMAO levels were observed over a 4-week interval in individuals consuming a high-fatty diet compared to individuals consuming a low fatty diet [75]. Thus, a Western diet may increase circulating TMAO levels leading to cardiac inflammation and fibrosis, contributing to cardiac dysfunction. Dietary L-carnitine chronic supplementation could accelerate CAD by altering the microbial composition [45]. A recent human study showed that omnivore volunteers significantly generate more TMAO than vegans/vegetarians from oral L-carnitine because of marked increase in gut microbial conversion of γBB into TMA [72]. Thus, gut microbiota γBB→TMA/TMAO transformation may induce by omnivorous dietary patterns and chronic l-carnitine exposure [72]. A recent study of 345 adults found that in adults with SCA compared with those without SCA, higher levels of *Escherichia coli* were shown as well as an increased TMAO production [70]. *E. coli* also correlated with increased LPS absorption and macrophage activation. Gut-derived LPS from *E. coli* that localizes in human carotid plaque has a potential role as a pro-inflammatory molecule in the atherosclerotic lesion [87]. In mice, compared with the control diet, a diet supplemented with choline enhanced the production of TMAO exacerbating pressure overload-induced heart failure organ [86]. In humans, ingested dietary phosphatidylcholine (lecithin) may also serve as fuel for gut microbiota enhancing the production of TMAO.

On the other hand, the Mediterranean diet is well known to be an optimal dietary prevention of cardiovascular events [71,88]. Specifically, greater adherence to a Mediterranean diet showed a beneficial role on reducing TMAO levels [71]. Moreover, a high-fiber diet increased SCFA levels, including levels of acetate. Interestingly, dietary intake of fiber and supplementation with acetate could modulate cardiac molecular pathways beneficial for cardiovascular function, lowered blood pressure, decreased cardiac hypertrophy and fibrosis, and improved heart function in experimental hypertension [76].

The Mediterranean diet is also rich in fish and flaxseed oil, which have a role in reducing TMAO levels, promoting proliferation of SCFA-producing bacteria, and inhibiting those producing LPS in the gut. A similar effect is also provided by polyphenols—a group of phytochemicals—and beta-glucan, which also reduces blood levels of glucose and cholesterol, providing protection against CVD and diabetes. Both of these molecules abound in the Mediterranean diet [89].

## 10. Probiotics, Microbiota and CAD

As shown by several studies, probiotics exert a protective effect against CAD, mainly through a positive impact over all the main risk factors for atherosclerosis. Recent evidence, in fact, points out that regular consumption of probiotics would provide beneficial effects in lowering low density lipoprotein (LDL) cholesterol, blood pressure, inflammatory mediators, blood glucose levels, and body mass index [90].

The composition of intestinal microbiota has been shown to be altered in patients affected by type 2 Diabetes Mellitus and it seems to have a role in inducing insulin-resistance [91]. Intake of probiotics—particularly those belonging to *Lactobacillus* and *Bifidobacterium*—induce a positive remodulation of gut microbiota that leads to a more resistant intestinal barrier. This stops LPS from translocating to the systemic circulation, thus reducing the release of pro-inflammatory cytokines, as well as glycosylated hemoglobin A1c (HbA1c). At the same time, the correction of dysbiosis promoted by probiotics improves the production of saccharolytic fermentation and SCAFs, which are implicated in the release of glucagonal peptide-1 (GLP-1), a molecule that increases insulin sensitivity [92]. A review on the metabolic effects of probiotics shows how they provide beneficial effects on obesity and insulin resistance, as well as on carbohydrate metabolism, fasting blood glucose and antioxidant status. The authors were also able to find studies that underlie how probiotics and symbiotics improve liver metabolism in patients suffering from non-alcoholic fatty liver disease (NAFLD) [93].

Even dyslipidaemia—another major risk factor for CAD—is positively affected by regular consumption of probiotics. Indeed, with a review focused on the hypocholesterolaemic effect of probiotics, Reis, De Rosa, and Peluzio identify the most studied molecular mechanisms through which they are able to provide a significant improvement in the serum lipid profile. The main ones seem to include deconjunction of bile salts and reduced absorption of intestinal cholesterol, incorporation of cholesterol within the probiotic cell membrane, conversion of cholesterol into coprostanol, and inhibition of hepatic cholesterol synthesis [94]. Despite the fact that all of these mechanisms are still poorly understood, the role of probiotics in reducing bile acid reabsorption and in inhibiting intestinal cholesterol absorption is confirmed by a more recent review by Hassan et al. [95].

Hence, probiotics may soon become a pivotal therapy in preventing CAD, but further studies are needed to assess the effective role of these supplements in inhibiting the developments of atherosclerosis, as well as to identify the specific strains that would test better. Indeed, so far, the choice of the probiotic candidate strain has been almost always empirical. To remedy this, Jiang et al. made an effort to test a specific human-derived *Lactobacillus mucosae A1* that was shown to be associated with a high-fiber diet through a metagenomic analysis. The study shows that supplementation with *Lactobacillus mucosae A1* in mice on a Western diet attenuates severe lipid accumulation in serum, liver and aortic serum. Despite the fact that the molecular mechanism through which this particular strain provides such an effect is still unclear, the study suggests that diet implementation with *Lactobacillus mucosae A1* is effective in treating hyperlipidemia and atherosclerosis in mice [96].

## 11. Conclusions

The literature gathered in our review points out how gut microbiota is closely connected to coronary artery disease, and all the studies confirm a different composition between CAD patients and the healthy population. The latest evidence has shown that this connection is mainly dependent on TMAO, SCFAs and LPS which influence atherosclerosis through metabolic and inflammatory effects. Although microbiota analysis could be potentially useful in predicting cardiovascular risk, the lack of uniformity of results prevents us from using it in clinical practice. One of the reasons for this lack of homogeneity may be the fact that each segment of the GI tract has been shown to have a very peculiar microbiota composition [97], making it even more difficult to identify unique clusters of pathogenic and nonpathogenic microorganisms.

In addition, studies that explore the link between the microbiota and acute coronary syndrome are still scarce, hence we are far from using this analysis in emergency departments as a diagnostic marker. If further expanded, the data we now have available may have mostly a prognostic value, for example, through the identification of clusters associated with classes of risk for CAD.

Moreover, studies about the association between gut microbiota and pollution show that the gut microbiota homeostasis, which depends on various factors including pollution and lifestyle, is related to cardiovascular risk.

In this setting, modulation of gut microbiota composition in patients with CAD through dietary intervention and probiotics represents a promising therapeutic target. Hence, the modulation of the gut microbiota through lifestyle, treatment and medications could potentially prevent and treat cardiovascular disease.

## Figures and Tables

**Table 1:** Studies on gut microbiota composition in CAD patients.

Authors	Study Design	Year	Subjects	Findings
Fialho A. et al. [23]	Observational	2018	160 patients who underwent coronary angiography	Patients with SIBO had a higher frequency of CAD
Karlsson F.H. et al. [24]	Comparative	2012	12 patients with symptomatic atherosclerotic plaques and 13 healthy patients	Symptomatic atherosclerosis is associated with *Collinsella*. *Eubacterium* and *Roseburia* are predominant in the control group
Emoto T. et al. [25]	Comparative	2016	39 CAD patients and 50 healthy volunteers	CAD patients have an increase in Lactobacillales and a reduction of phylum Bacteroidetes
Zhu Q. et al. [26]	Comparative	2018	70 CAD patients and 98 healthy controls	CAD patients have an increase in *Faecalibacterium*, *Subdoligranum*, *Roseburia* and *Eubacterium* and a reduction of *Escherichia*, *Shigella* and *Enterococcus*
Kelly T.N. et al. [27]	Long-term epidemiologic	2016	112 participants	Participants who developed a high cardiovascular disease risk have an increase in *Prevotella* and *Tyzzerella*
Li J. et al. [28]	Comparative	2017	155 patients with pre-hypertension or primary hypertension and 41 healthy controls	Patients with primary hypertension have an increase in *Prevotella* and *Klebsiella* and a reduction of *Faecalibacterium*, *Oscillibacter*, *Roseburia*, *Bifidobacterium*, *Coprococcus*, and *Butyrivibrio*
Cui L. et al. [29]	Comparative	2017	29 CAD patients and 35 healthy controls	CAD patients have an increase in phyla Firmicutes and Fusobacteria and a reduction of Bacteroidetes and Proteobacteria
Yoshida N. et al. [30]	Comparative	2018	30 CAD patients and 30 healthy controls	CAD patients have a reduction of *Bacteroides vulgatus* and *Bacteroides dorei*. Treatment with with these two species attenuated atherosclerotic lesion formation in atherosclerosis-prone mice
Toya T. et al. [31]	Comparative	2020	53 advanced CAD patients and 53 healthy controls	CAD patients have an increase in *Ruminococcus gnavus* and a reduction of *Lachnospiraceae NK4B4* and *Ruminococcus gauvreauii*

Abbreviations: CAD, coronary artery disease.

**Table 2:** Studies on the association between TMAO and cardiovascular disease.

Authors	Type	Year	Subjects	Findings
Stubbs J.R. et al. [32]	Prospective	2016	220 patients with chronic kidney disease who underwent coronary angiography	Increased TMAO concentrations correlate with coronary atherosclerosis burden
Li X.S. et al. [33]	Metabolomics analyses	2018	CAD patients and healthy controls	The levels of trimethyllysine, a nutrient precursor of TMAO, are associated with major adverse cardiovascular event risks
Lent-Schochet D. et al. [34]	Comparative	2018	30 patients with metabolic syndrome and 20 healthy controls	TMAO levels increase in metabolic syndrome
Randrianarisoa E. et al. [35]	Prospective	2016	220 patients	TMAO levels are positively associated with visceral fat mass and liver fat content and negatively associated with insulin sensitivity and carotid intima-media thickness
Haghikia A. et al. [36]	Prospective	2018	Two prospective cohorts of patients with first-ever ischemic stroke	TMAO levels are linked with an increased risk of myocardial infarction, recurrent stroke and cardiovascular death
Matsuzawa Y. et al. [37]	Observational	2019	112 STEMI patients	TMAO levels are a significant and independent predictor of future cardiovascular events in patients after STEMI
Li X.S. et al. [38]	Multicenter	2019	530 and 1053 patients with suspected ACS	TMAO levels are associated with both near- and long-term CV events in patients with chest pain and ACS
Gao J. et al. [39]	Prospective	2020	30 patients with unstable angina pectoris, 30 post-STEMI and 30 healthy controls	Elevated serum TMAO levels correlated with ACS
Tascanov M.B. et al. [40]	Prospective	2020	44 young patients with ACS (<50 years of age), 39 elderly patients with ACS, and 44 healthy control	Young patients with ACS had significantly higher levels of TMAO compared to the control and elderly ACS groups
Seldin M.M. et al. [41]	Prospective	2016	Mice with chronic dietary TMAO supplementation	TMAO activates inflammatory pathways in cells of the vasculature, leading to atherosclerosis
Ma G. et al. [42]	Prospective	2017	Mice with chronic dietary choline supplementation	TMAO promotes cell adhesion molecule-1 (VCAM-1) expression and monocyte adherence
Chen M.L. et al. [43]	Prospective	2017	Mice with chronic dietary choline supplementation	TMAO promotes vascular inflammation by activating the NLRP3 inflammasome
Geng J. et al. [44]	Prospective	2018	Mice with chronic dietary TMAO supplementation	TMAO promotes the atherosclerosis via CD36/MAPK/JNK pathway
Koeth R.A. et al. [45]	Prospective	2013	Mice with chronic dietary L-carnitine supplementation	L-carnitine produces TMAO that modulates cholesterol and sterol metabolism and increases atherosclerosis
Wang et al. [46]	Prospective	2011	Mice with chronic dietary choline, TMAO or betaine supplementation	TMAO promotes the up regulation of multiple macrophage scavenger receptors linked to atherosclerosis
Chen M.L. et al. [47]	Randomized controlled trial	2016	Mice treated with resveratrol	Resveratrol decreases TMAO levels and increases hepatic biliar acids neosynthesis
Ding L. et al. [48]	Prospective	2018	Mice with chronic dietary TMAO supplementation	TMAO accelerates aortic lesion formation in mice by altering bile acid profiles
Zhu W. et al. [49]	Prospective	2016	Mice with chronic dietary TMAO supplementation	TMAO directly enhances human platelet responsiveness
Skye S.M. et al. [50]	Randomized controlled trial	2018	Mice transplanted with human microbiota from a low or high TMAO donor	Humanized mouse with high TMAO microbiome has an increased platelet reactivity and thrombosis potential
Shih D.M. et al. [51]	Prospective	2019	FMO3 knockout mouse	FMO3 knockout mice have a reduction of TMAO accompanied by decreased platelet responsiveness
Roberts A.B. et al. [52]	Prospective	2018	Mice with chronic dietary choline supplementation	Inhibition of gut microbial TMA and TMAO production reduces thrombosis potential

Abbreviations: ACS, acute coronary syndrome; CAD, coronary artery disease; CV, cardiovascular; FMO3, Flavin Containing Dimethylaniline Monoxygenase 3; STEMI, ST-Elevation Myocardial Infarction; TMA, trimethylamine; TMAO, trimethylamine-N-oxide.

**Table 3:** Studies on the association between SCFAs and cardiovascular disease.

Authors	Type	Year	Subjects	Findings
McNelis J.C. et al. [53]	Prospective	2015	GPR43 knockout mice	SCFA/GPR43 system can regulate the process of β-cell compensation in the insulin-resistant state
den Besten G. et al. [54]	Prospective	2015	Mice with chronic dietary SCFA supplementation	SCFAs protect against obesity and improved insulin sensitivity through downregulation of PPARγ
Vinolo M.A. et al. [55]	Prospective	2011	Mice with chronic dietary SCFA supplementation	SCFAs inhibit production of proinflammatory cytokines by LPS-stimulated neutrophils
Aguilar E.C. et al. [56]	Prospective	2014	Mice with chronic dietary butyrate supplementation	Butyrate can slow the progression of atherosclerosis by reducing adhesion and migration of macrophages and increasing plaque stability.

Abbreviations: GPR43, G-protein-coupled receptor 43; SCFA, short chain fatty acid.

**Table 4:** Studies on the association between LPS and cardiovascular disease.

Authors	Type	Year	Subjects	Findings
Ostos M.A. et al. [57]	Prospective	2002	ApoE-deficient mice injected with LPS	LPS administration aggravates atherosclerosis in apoE-deficient mice
Geng S. et al. [58]	Prospective	2016	ApoE-deficient mice injected with LPS	LPS causes significant elevation of pro-inflammatory cytokines such as TNF-α and IL-6
Zhou X. et al. [59]	Comparative	2018	50 CAD patients, 50 STEMI patients and 49 healthy control	LPS in STEMI patients was significantly increased maybe for a gut bacterial translocation into systemic circulation
Kasahara K. et al. [60]	Prospective	2017	ApoE-deficient mice	ApoE− deficient mice are resistant to the development of atherosclerosis and this is associated with a reduction of LPS
Fuijkschot W.W. et al. [61]	Prospective	2018	ApoE-deficient mice injected with LPS	LPS injection triggers a systemic inflammation, but does not increase atherosclerotic plaque area or inflammatory cell density
Anzulovic-Mirosevic D. et al. [62]	Comparative	2011	37 patients with left ventricular dysfunction, 7 acute myocardial infarction and 29 healthy controls	LPS levels were lower in patients with a chronic left ventricular dysfunction but without reaching any statistical significance

Abbreviations: CAD, coronary artery disease; LPS, lipopolysaccharides; STEMI, ST-Elevation Myocardial Infarction.

**Table 5:** Studies on the association between pollution, gut microbiota and cardiovascular disease.

Authors	Type	Year	Subjects	Findings
Zhang S. et al. [65]	Observational	2015	Mice exposed to low doses of Cd	Cadmium exposure alters the gut microflora composition and increases LPS production
Li X. et al. [66]	Prospective	2019	Mice treated with Cadmium or Arsenic	Cadmium and arsenic cause a depletion of Bacteroides and affect bile acids production
Jin C. et al. [67]	Prospective	2018	Mice with chronic exposure to carbendazim	Chronic carbendazim exposure induces gut microbiota dysbiosis and disturbs lipid metabolism
Mutlu E.A. et al. [68]	Prospective	2018	Mice exposed to particulate matters	Exposure to particulate matters alters gut microbiota and induces inflammation in the GI tract
Fitch M.N. et al. [69]	Prospective	2020	Mice exposed to wood-smoke (WS) and mixed diesel and gasoline vehicle exhaust (MVE)	Inhalation exposure to WS or MVE alters gut microbiota and increases markers of an inflammatory response

**Table 6:** Studies on the association between diet, gut microbiota and cardiovascular disease.

Authors	Type	Year	Subjects	Findings
Baragetti et al. [70]	Observational	2021	345 adults with or without subclinical carotid atherosclerosis (SCA)	*Faecalibacterium prausnitzii* in the absence of SCA and *Escherichia coli* in the presence of SCA are directly related to over-representation of metagenomic pathways linked to different dietary sources: - Sulfur oxidation and starch degradation in absence of SCA; - Metabolism of amino acids, syntheses of palmitate, choline, carnitines and TMAO in presence of SCA
De Filippis et al. [71]	Observational	2016	153 Italian healthy adults (51 vegetarians, 51 vegans and 51 omnivores)	- Habitual vegetarian and vegan diets promote enrichment of fibre-degrading bacteria in the gut; - Subjects who consume a Mediterranean diet rich in fruit, legumes and vegetables have higher levels of SCFAs; - Low adherence to the Mediterranean diet corresponds to an increase in urinary TMAO levels, a potential risk factor for cardiovascular disease.
Koeth et al. [45]	Mice model	2013	Mice on either normal chow (*n* = 10) or carnitine supplemented diet (*n* = 11)	Chronic dietary L-carnitine supplementation significantly altered microbial composition, markedly enhanced synthesis of TMA/TMAO, and increased atherosclerosis, but not following suppression of intestinal microbiota.
Koeth et al. [72]	Observational	2019	32 vegans/vegetarians volunteers and 40 omnivores volunteers	Dietary l-carnitine is converted into the atherosclerosis- and thrombosis-promoting metabolite TMAO via 2 sequential gut microbiota-dependent transformations: (a) initial rapid generation of the atherogenic intermediate γBB, followed by (b) transformation into TMA via low-abundance microbiota in omnivores, and to a markedly lower extent, in vegans/vegetarians.Gut microbiota γBB→TMA/TMAO transformation is induced by omnivorous dietary patterns and chronic l-carnitine exposure.
Tang et al. [73]	Prospective	2013	First study: 40 healthy adults ingesting deuterium-labeled phosphatidylcholine and two hard-boiled eggsSecond study: 4007 adults who underwent diagnostic cardiac catheterization	The production of TMAO from dietary phosphatidylcholine is dependent on metabolism by the gut microbiota. Pathways that are dependent on the gut microbiota may contribute to the pathophysiology of atherosclerotic CAD.
Organ et al. [74]	Controlled mice model	2016	Mice fed either a control diet, a diet containing choline, a diet containing TMAO	Choline diet and its gut microbiota derived metabolite, TMAO, exacerbate pressure overload-induced hear failure.
Park et al. [75]	Randomized, crossover study	2019		Elevated TMAO levels were observed over a 4-week interval in individuals consuming a high-fat diet (HFD) that is predominantly animal based, compared to individuals consuming a low fat and the MD
Marques et al. [76]	Controlled mice model	2017	Mice fed with a control diet or high-fiber diet, or acetate supplementation.	Fiber and acetate changed the gut microbiota composition, increasing the prevalence of acetate-producing bacteria, improving the levels of *Bacteroides acidifaciens*, and decreasing gut dysbiosis.Dietary intake of fiber and supplementation with acetate modulated renal and cardiac molecular pathways beneficial for cardiovascular function, lowered blood pressure, decreased cardiac hypertrophy and fibrosis, and improved heart function in experimental hypertension.
